# Supplementary material for: Surface Modification and Subsequent Fermi Density Enhancement of Bi(111)
Source: J Phys Chem C Nanomater Interfaces. 2021 Mar 5;125(10):5549–58. doi: 10.1021/acs.jpcc.0c07345 (PMC8279637; doi:10.1021/acs.jpcc.0c07345)
Supplement: Supplementary file 1 — jp0c07345_si_001.pdf [file jp0c07345_si_001.pdf]

Supporting Information

Surface Modification and Subsequent Fermi Density Enhancement of Bi(111)

Kuanysh Zhussupbekov,[*,†] Killian Walshe,[†] Brian Walls,[†] Andrei Ionov,[‡] Sergei I. Bozhko,[‡] Andrei Ksenz,[‡] Rais N. Mozhchil,[‡] Ainur Zhussupbekova,[†] Karsten Fleischer,[¶] Samuel Berman,[†] Ivan Zhilyaev,[§] David D. O'Regan,[*,†] and Igor V. Shvets[*,†]

†*School of Physics and Centre for Research on Adaptive Nanostructures and Nanodevices (CRANN), Trinity College Dublin, Dublin 2, Ireland*

‡*Institute of Solid State Physics, Russian Academy of Sciences, Chernogolovka, Russia*

¶*School of Physical Sciences, Dublin City University, Dublin 9, Ireland*

§*Institute of Microelectronics Technology and High Purity Materials, Russian Academy of Sciences, Chernogolovka, Russia*

‖*AMBER, the SFI Research Centre for Advanced Materials and BioEngineering Research, Ireland*

E-mail: zhussupk@tcd.ie; david.o.regan@tcd.ie; ivchvets@tcd.ie

Calculated and experimental LDOS.

Figure S1 represents dI/dV curves corresponding to the experimental bilayer terraces and calculated LDOS of the monolayer and bilayer terraces. Issues with tip stability, as a result of scanning rough sputtered surface, limited the quality of STS measurements, and hence, no dI/dV experimental results are presented for the monolayer. DFT calculations for the electron density at a distance of 3 Å from the surface termination for both bilayer and monolayer steps have been calculated. The bilayer calculation shows a good agreement with the corresponding STS data in the vicinity of the Fermi level with the minima shifted to the right of the Fermi level. The monolayer is calculated to have a larger LDOS in the vicinity of the Fermi level.

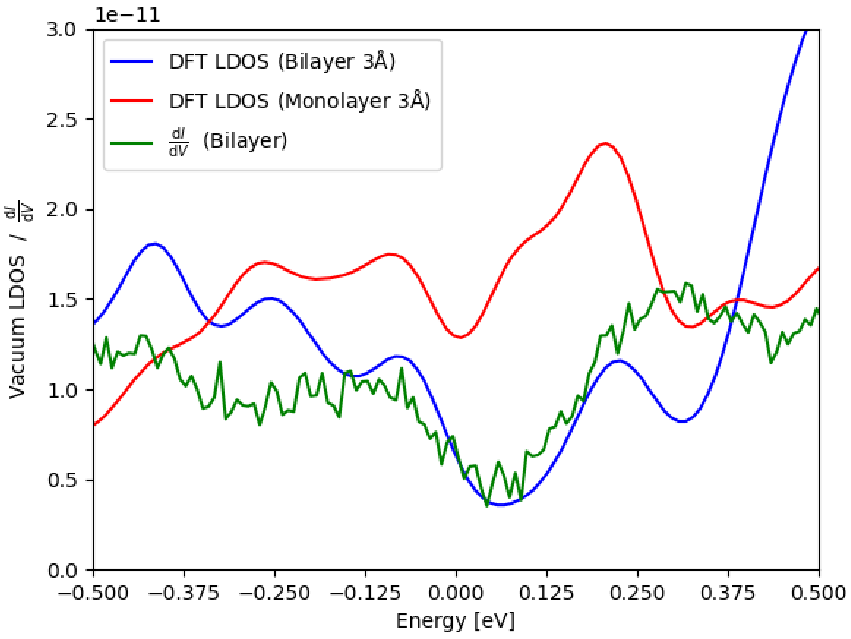

Figure S1: Calculated and experimental LDOS. LDOS calculations for both bilayer and monolayer terraces were performed at a distance of 3 Å

LEED analysis prior and after Ar^+ sputtering

Spot profiles analysis of the LEED pattern before and after Ar^+ treatments were performed.[1] We have analysed our LEED images which were taken at the same settings. Since we have 2 LEED

systems employed at different temperatures, we can compare LEED images before and after Ar^+ bombardment separately at room temperature and at low temperature. Figure S2 demonstrates spot profiles prior and after Ar^+ etching at room and low (110K) temperatures. In case of the room temperature measurement it can be seen that there is very small deviation between spots (black:before and red:after treatment).

In case of the low temperature LEED measurement the spot intensity slightly decreased after Ar^+ etching (black:before and red:after treatment). It should be noted that in this particular LEED system we have a cylindrical sample holder which can rotate. That is why we measured intensity of the brightest spot. Bi single crystal was glued exactly in the centre of this sample holder.

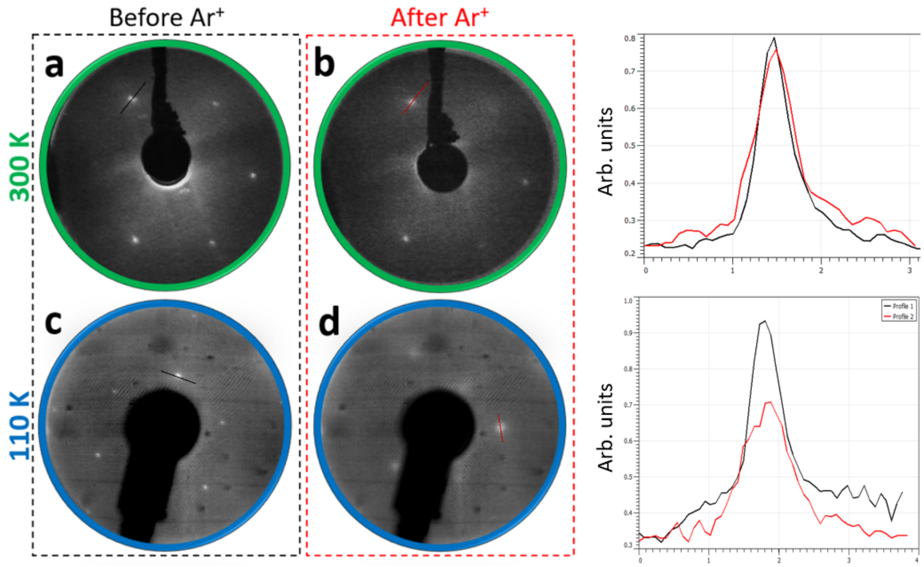

Figure S2: Spot profiles of the LEED measurements before and after Ar^+ bombardment.

XPS analysis after Ar^+ sputtering

Figure S3 is the XPS spectra obtained after Ar^+ sputtering. From the Carbon 1s, Oxygen 1s and Argon 2p regions it is clear that no contamination is present on the surface. This XPS analysis could exclude a possibility of surface contamination. Also, taking into account relatively low sputtering energy (2keV) and absence of the Argon on the XPS spectra it can be concluded that no Ar^+ ion implantation induced by Ar^+ sputtering.[2–4]

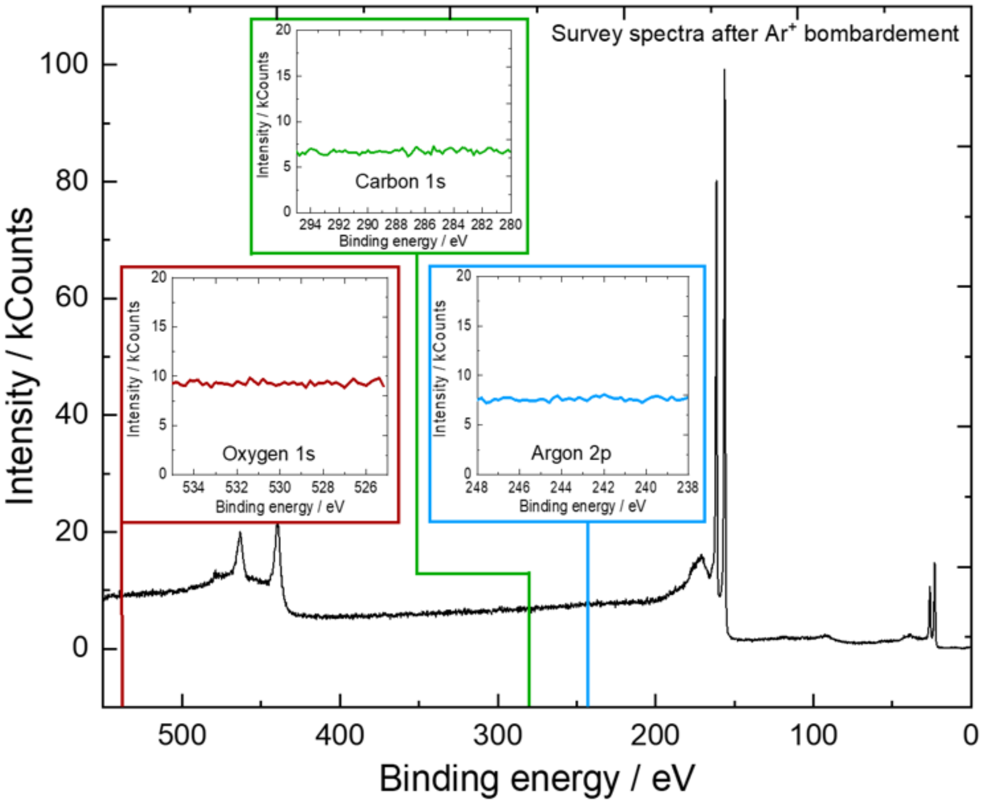

Figure S3: Survey and zoomed XPS scans of the Carbon 1s, Oxygen 1s and Argon 2p regions after Ar^+ ion sputtering, which indicates no signature of the contamination and Argon on the surface.

Integrated LDOS

In Figure S4, we present the integrated LDOS plots of the surface region atoms of the monolayer terrace structure from the Fermi level to ±1 eV.

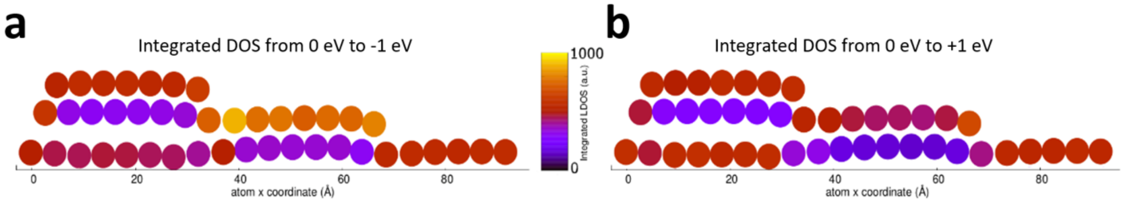

Figure S4: Integrated plots of calculated LDOS. **(a)** Integrated LDOS plot from the Fermi level to -1 (eV). **(b)** Integrated LDOS plot from the Fermi level to +1 (eV).

References

(1) Tanuma, S.; Powell, C. J.; Penn, D. R. Calculations of electorn inelastic mean free paths. II. Data for 27 elements over the 50-2000 eV range. *Surface and Interface Analysis* **1991**, *17*, 911–926.

(2) Donya, H.; Salah, A. Effect of 60 keV argon ion implantation in Makrofol® DE 1-1 on the optical properties. *Polymer Bulletin* **2019**, *77*, 6349–6375.

(3) Lacquet, B.; Swart, P. Argon ion implantation gettering of large area p-n junctions and schottky diodes. *Nuclear Instruments and Methods in Physics Research Section B: Beam Interactions with Materials and Atoms* **1985**, *6*, 372–375.

(4) Sanders, I.; Williams, B.; Smith, B.; Stephen, J.; Hinder, G. The effect of argon implantation on the conductivity of boron implanted silicon. *Solid-State Electronics* **1977**, *20*, 703–707.
